# Tuberculostearic Acid Controls Mycobacterial Membrane Compartmentalization

**DOI:** 10.1128/mbio.03396-22

**Published:** 2023-03-28

**Authors:** Malavika Prithviraj, Takehiro Kado, Jacob A. Mayfield, David C. Young, Annie D. Huang, Daisuke Motooka, Shota Nakamura, M. Sloan Siegrist, D. Branch Moody, Yasu S. Morita

**Affiliations:** a Department of Microbiology, University of Massachusetts, Amherst, Massachusetts, USA; b Division of Rheumatology, Inflammation and Immunity, Brigham and Women’s Hospital, Harvard Medical School, Boston, Massachusetts, USA; c Research Institute for Microbial Diseases, Osaka University, Suita, Osaka, Japan; d Molecular and Cellular Biology Graduate Program, University of Massachusetts, Amherst, Massachusetts, USA; Weill Cornell Medicine

**Keywords:** fatty acids, lipidomics, membrane domain, phospholipids, transposon sequencing

## Abstract

The intracellular membrane domain (IMD) is a laterally discrete region of the mycobacterial plasma membrane, enriched in the subpolar region of the rod-shaped cell. Here, we report genome-wide transposon sequencing to discover the controllers of membrane compartmentalization in Mycobacterium smegmatis. The putative gene *cfa* showed the most significant effect on recovery from membrane compartment disruption by dibucaine. Enzymatic analysis of Cfa and lipidomic analysis of a *cfa* deletion mutant (Δ*cfa*) demonstrated that Cfa is an essential methyltransferase for the synthesis of major membrane phospholipids containing a C_19:0_ monomethyl-branched stearic acid, also known as tuberculostearic acid (TBSA). TBSA has been intensively studied due to its abundant and genus-specific production in mycobacteria, but its biosynthetic enzymes had remained elusive. Cfa catalyzed the *S*-adenosyl-l-methionine-dependent methyltransferase reaction using oleic acid-containing lipid as a substrate, and Δ*cfa* accumulated C_18:1_ oleic acid, suggesting that Cfa commits oleic acid to TBSA biosynthesis, likely contributing directly to lateral membrane partitioning. Consistent with this model, Δ*cfa* displayed delayed restoration of subpolar IMD and delayed outgrowth after bacteriostatic dibucaine treatment. These results reveal the physiological significance of TBSA in controlling lateral membrane partitioning in mycobacteria.

## INTRODUCTION

Mycobacteria are encapsulated by a thick and waxy cell envelope, which acts as a barrier to antibiotics and host immunity. Mycobacterial cell envelopes are worthy of investigation because of the high worldwide burden of infectious diseases caused by mycobacteria. Also, mycobacterial membranes have evolved structural adaptations that are fundamentally different from eukaryotic cells and model eubacterial organisms based on the number and nature of cell envelope layers present. The mycobacterial cell envelope consists of five biochemically distinct layers: the plasma membrane, the peptidoglycan layer, the arabinogalactan layer, the mycomembrane, and the capsule (1–4). The high impermeability of the mycobacterial cell envelope is attributed to long-chain mycolic acids of the outer mycomembrane. However, the inner plasma membrane may also contribute to the regulation of the permeability of mycobacterial cellular response (5). Indeed, our recent study suggests that mycobacteria have a rapid stress response mechanism to remodel the plasma membrane after exposure to membrane-fluidizing chemicals (6). The main building blocks of the plasma membrane are phospholipids, of which cardiolipin, phosphatidylethanolamine (PE), phosphatidylinositol (PI), and PI mannosides (PIMs) are the major phospholipid species. Fine-tuning the composition of phospholipid headgroups and hydrocarbon chains ensures the overall integrity of the plasma membrane (7).

Further, recent work makes clear that the mycobacterial plasma membrane is laterally heterogenous and actively regulated. Density gradient fractionation of cell lysate revealed two physically separable fractions with distinct densities and content. One, containing plasma membrane free of cell wall components, is called the intracellular membrane domain (IMD). A second denser fraction, containing plasma membrane tightly associated with cell wall components, is called the PM-CW (8, 9). The proteome and lipidome of the IMD are distinct from those of the PM-CW; the IMD harbors enzymes that are important for active growth and homeostasis, suggesting that the IMD is a biosynthetic hub in the bacteria (8). Consistent with a role in active growth, the IMD is enriched in the subpolar region, where rod-shaped mycobacteria grow and elongate (10–12).

In nongrowing stationary-phase cells, the IMD is spatially reorganized and delocalized from the subpolar regions to more proximal columnar parts of the cell (13). IMD delocalization was also observed under nutrient starvation and upon cell wall-targeted antibiotic treatment. Furthermore, membrane-targeted perturbations induced by fluidizers such as benzyl alcohol and dibucaine disrupted the subpolar enrichment of the IMD (14, 15). Thus, emerging data suggest that mycobacteria have a mechanism to spatiotemporally coordinate the IMD in response to stress and different growth conditions.

To gain insight into the molecular mechanism of plasma membrane partitioning in mycobacteria, we screened for genes that are critical for Mycobacterium smegmatis to remain viable during and recover from dibucaine treatment. Dibucaine is a topical anesthetic which preferentially inserts into the liquid-ordered phase of a membrane to fluidize the membrane and perturb the lateral membrane organization (16). Here, we used unbiased whole-organism genetic and lipidomic screens and targeted gene deletion to discover that cyclopropane-fatty-acyl-phospholipid synthase (encoded by *cfa*) controls the membrane recovery response and mycobacterial growth. Mechanistic investigation demonstrates that Cfa plays an essential role in producing an abundant and Mycobacterium-characteristic lipid known as tuberculostearic acid (TBSA) that is distributed among the major membrane phospholipids and controls membrane compartmentalization and growth of M. smegmatis.

## RESULTS

### Dibucaine is bacteriostatic and transiently delocalizes IMD from the subpolar regions.

**FIG 1:**
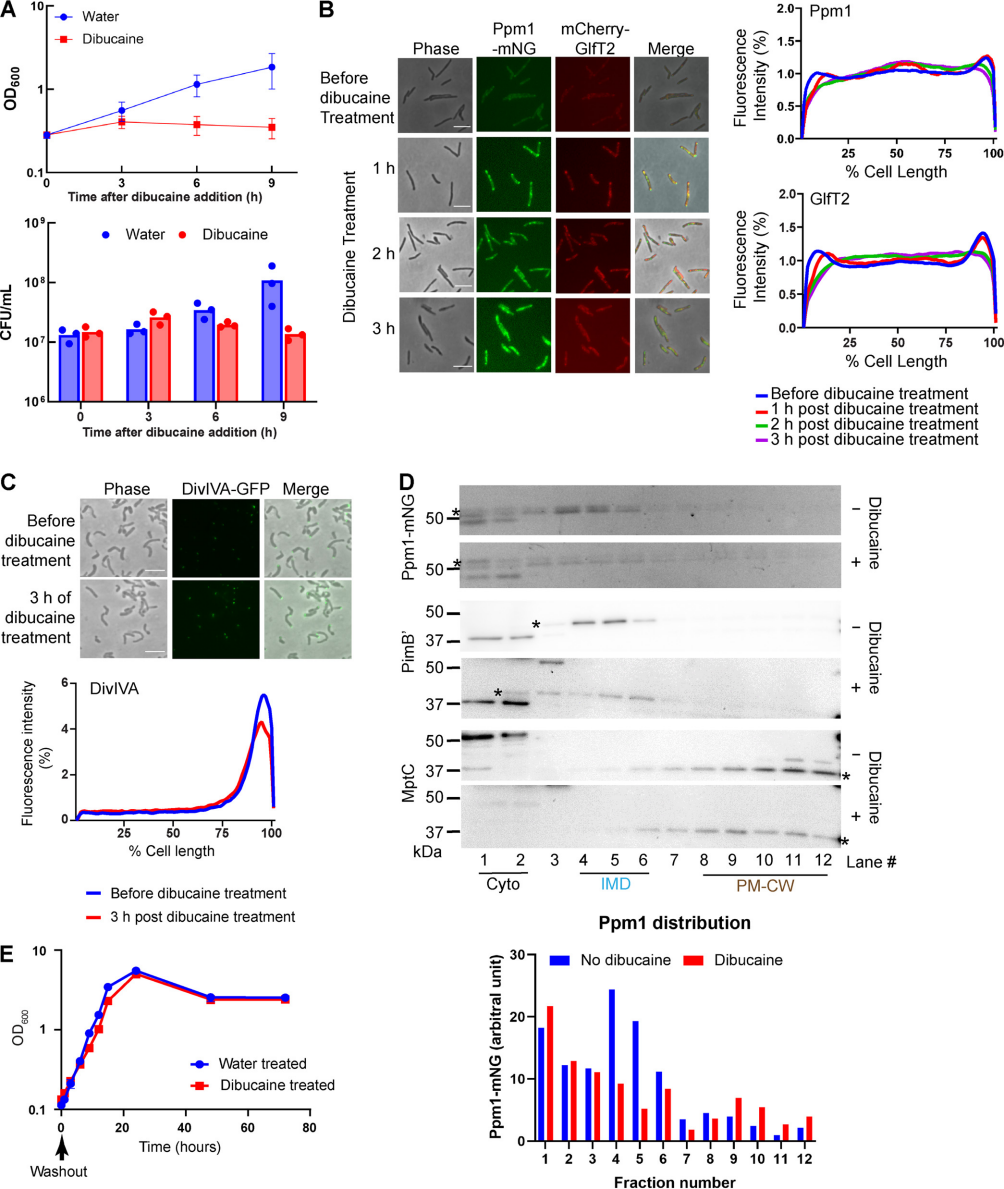
Dibucaine specifically delocalizes the subpolar enrichment of IMD proteins in M. smegmatis. (A) Cells expressing mCherry-GlfT2 and Ppm1-mNeonGreen from the endogenous loci were incubated with or without dibucaine for 9 h, and the OD_600_ and CFU were determined every 3 h in biological triplicates. Averages and standard deviations or individual data points are shown. (B) Effect of dibucaine on IMD-associated proteins. IMD-marker proteins, mCherry-GlfT2 and Ppm1-mNeonGreen, were observed every hour during dibucaine treatment (left). Representative images from biological triplicates are shown. Scale bar = 5 μm. Fluorescence intensity profiles along the long axis of the cells were quantified using Oufti (right). Ppm1: *n* = 237 (before), 121 (1 h), 289 (2 h), and 172 (3 h). GlfT2: *n* = 127 (before), 119 (1 h), 287 (2 h), or 169 (3 h). (C) The polar localization of DivIVA, a PM-CW marker protein, was examined before and after dibucaine treatment. Representative images from biological duplicates are shown. *n* = 36 (before) or 37 (3 h). (D) Sucrose gradient fractionation of cell lysates of the strain expressing mCherry-GlfT2 and Ppm1-mNeonGreen. The strain was treated with or without dibucaine. Ppm1-mNeonGreen (indicated by asterisks) was visualized by in-gel fluorescence after SDS-PAGE. The fluorescence intensity of each band was quantified and shown in a bar graph. Anti-PimB′ antibody sometimes detects a cytoplasmic protein migrating slightly lower than PimB′, and we do not know the nature of this protein (8). PimB′ and MptC (indicated by asterisks) were visualized by Western blotting using rabbit anti-PimB′ and anti-MptC antibodies. PimB′, an IMD marker; MptC, a PM-CW marker, which was unaffected by dibucaine treatment. Representative results from biological duplicates are shown. (E) Recovery from dibucaine treatment. The same strain expressing mCherry-GlfT2 and Ppm1-mNeonGreen was treated with dibucaine for 3 h, washed, and recovered in a fresh Middlebrook 7H9 medium. The growth recovery was monitored by OD_600_. Time 0 corresponds to the beginning of the recovery period. Representative results from biological duplicates are shown.

In our previous study, using recombinant M. smegmatis strains expressing IMD-associated proteins such as MurG-Dendra2, mCherry-GlfT2, and Ppm1-mNeonGreen, we demonstrated that the treatment of M. smegmatis with 200 μg/mL dibucaine for 3 h disrupts the subpolar localization of these IMD-associated proteins (14, 15). Using a previously established strain, in which mCherry-GlfT2 and Ppm1-mNeonGreen are expressed from the endogenous loci (8), we first measured bacterial growth and IMD marker dispersion over time during dibucaine treatment. In a 9-h window, we observed no growth and no viability decline (Fig. 1A). Consistent with previous observations (14, 15), subpolar localizations of both mCherry-GlfT2 and Ppm1-mNeonGreen were progressively diminished over 3 h (Fig. 1B). DivIVA, a pole-associated non-IMD control membrane protein, was unaffected by dibucaine (Fig. 1C). Selective effects on known IMD markers suggested that dibucaine specifically disrupts the IMD.

Although the subpolar enrichment of IMD was diminished, the IMD was still biochemically detectable after sucrose density gradient fractionation (Fig. 1D). However, the distribution of IMD marker proteins, Ppm1-mNeonGreen and PimB′, became more diffuse, again suggesting IMD disruption. MptC, a PM-CW marker, was unaffected. We then used a 3-h dibucaine pulse followed by wash and chase to examine recovery by growth rate (optical density at 600 nm [OD_600_]). Dibucaine showed little effect on growth, and cells resumed a normal rate of growth almost immediately (Fig. 1E). These results show that dibucaine disrupts IMD polarization and attenuates cell replication, but these bacteriostatic effects dissipate when dibucaine is removed.

### Cfa protects against dibucaine stress.

**FIG 2:**
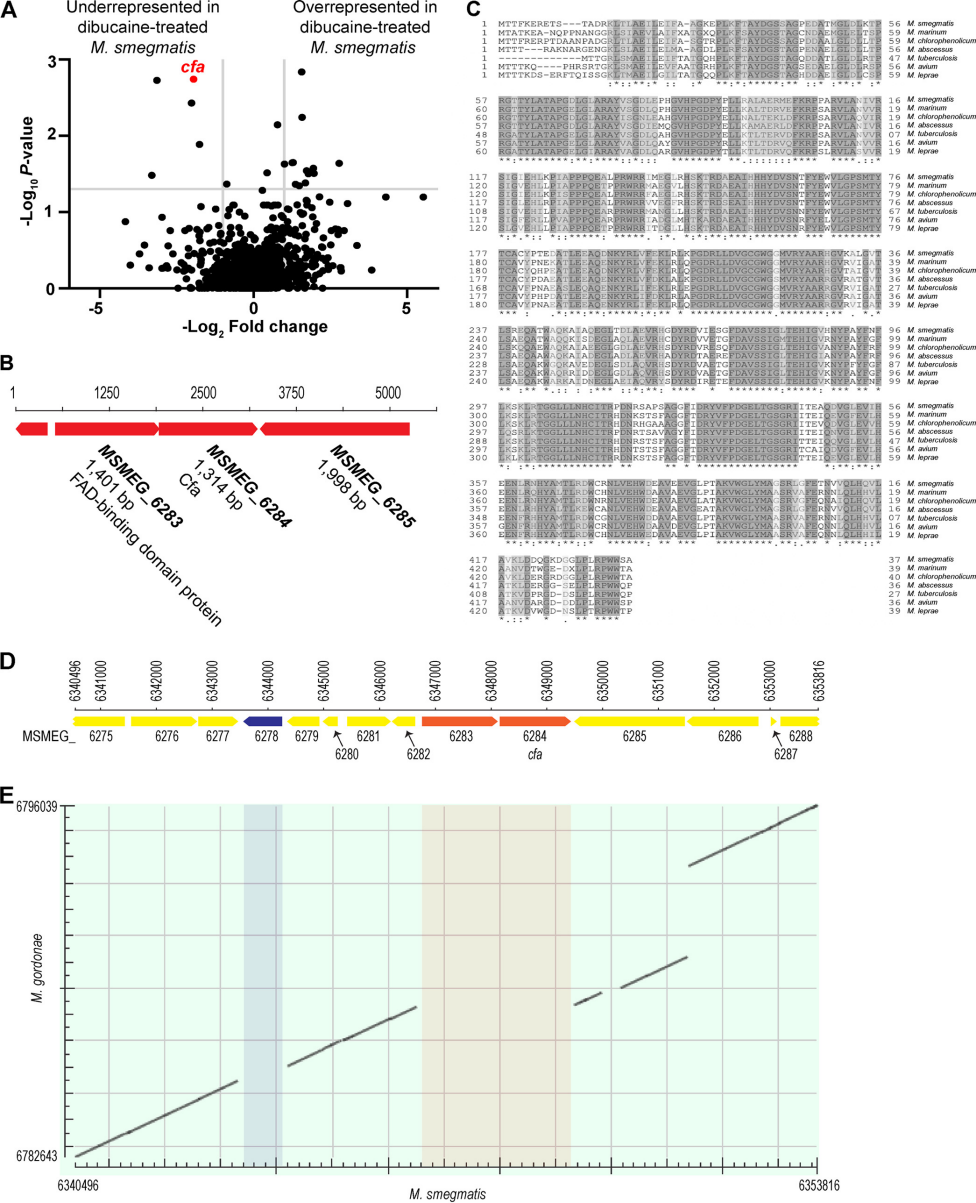
Tn-seq identifies *cfa* as a gene critical for surviving or recovering from dibucaine treatment. (A) The transposon mutant library was treated with dibucaine or water, washed, and recovered for ~16 h. Genes underrepresented or overrepresented in cells treated with dibucaine versus water are shown in the volcano plot. *P* values were calculated by Mann-Whitney U-test from 3 independent experiments. The horizontal gray line indicates *P* = 0.05; the vertical gray lines indicate 2-fold change. (B) The operon structure of *cfa* and the upstream gene encoding FAD-binding reductase in M. smegmatis. (C) Alignment of Cfa proteins. Cfa proteins from M. smegmatis mc^2^155, M. marinum, *M. chlorophenolicum*, M. abscessus, M. tuberculosis H37Rv, M. avium, and M. leprae were aligned using UniProt Align. (D) Map of the genome region surrounding the *cfa* gene, spanning region 6340496 to 6353816 in M. smegmatis (sequence ID: CP000480.1). (E) Pairwise alignment of the region 6340496 to 6353816 of M. smegmatis genome and the region 6782643 to 6796036 of M. gordonae genome (sequence ID: CP070973.1). The genome regions of M. smegmatis shaded in blue and brown correspond to MSMEG_6278 and MSMEG_6283-6284, respectively. These genes were missing from the syntenic region of the M. gordonae genome.

To discover the genes mediating resistance to dibucaine treatment, we treated a transposon (Tn) library of the M. smegmatis strain expressing mCherry-GlfT2 and Ppm1-mNeonGreen with dibucaine for 3 h. We compared Tn insertion frequencies in dibucaine-treated cells with those in vehicle-treated cells. The volcano plot showed five genes significantly diminished in frequency after dibucaine treatment (Fig. 2A, Table 1). Among them, *cfa* (*MSMEG_6284*) was depleted approximately 4-fold in dibucaine-treated cells in comparison to vehicle-treated cells, and it was the most significant genetic change detected (*P* value, 0.0018), suggesting that *cfa* is important for either surviving during or recovering from dibucaine treatment.

**TABLE 1:** Genes identified by Tn-seq

Gene locus	Log_2_ fold change	*P* value	Gene	Description
Underrepresented in dibucaine-treated M. smegmatis				
*MSMEG_5054*	−1.76	0.01301	*lpqG*	Probable lipoprotein
*MSMEG_5072*	−3.31	0.03296	*sigE*	RNA polymerase sigma-70 factor
*MSMEG_5488c*	−3.14	0.00188	*mprA*	DNA-binding response regulator
*MSMEG_6284*	−1.95	0.00181	*cfa*	Cyclopropane-fatty-acyl-phospholipid synthase
*MSMEG_6398*	−2.02	0.00373		Antigen 85-A
Overrepresented in dibucaine-treated M. smegmatis				
*MSMEG_0840c*	1.69	0.04153		Hypothetical protein
*MSMEG_1567*	1.73	0.02884		Hypothetical protein
*MSMEG_1886*	1.95	0.02661	*desA3*	NADPH-dependent stearoyl-CoA 9-desaturase
*MSMEG_2772*	1.57	0.00146		Amino acid permease
*MSMEG_3119*	2.79	0.02304		Unknown transporter
*MSMEG_3227*	1.37	0.04332	*pyk*	Pyruvate kinase
*MSMEG_4192c*	1.49	0.04533		Hypothetical protein
*MSMEG_4323*	1.58	0.00573		Pyruvate dehydrogenase E1 component
*MSMEG_5121c*	1.97	0.03121		Aminotransferase
*MSMEG_5672c*	1.29	0.02250	*gltA*	Type II citrate synthase
*MSMEG_5694*	1.79	0.03156		Hypothetical protein
*MSMEG_6761*	1.01	0.02351		Glycerol-3-phosphate dehydrogenase

The *cfa* gene encodes a putative *S*-adenosyl-l-methionine (SAM)-dependent methyltransferase involved in cyclopropane fatty acyl phospholipid synthesis and forms a putative operon with the upstream *MSMEG_6283* gene annotated as a flavin adenine dinucleotide (FAD)-binding domain protein (Fig. 2B). The *cfa* operon structure is widely conserved among mycobacteria, and Cfa is a highly conserved protein. For example, the amino acid identity of M. smegmatis Cfa to the ortholog in the key pathogen, Mycobacterium tuberculosis, is 78% while the identity to that in Mycobacterium leprae, which is likewise pathogenic and has otherwise undergone massive restriction in its genome size compared to other mycobacteria (17), is 74% (Fig. 2C). In Escherichia coli, heterologous expression of this operon from Mycobacterium chlorophenolicum resulted in the production of 10-methyl octadecanoic acid, also known as tuberculostearic acid (TBSA), from oleic acid (18). Moreover, one study reported the presence of TBSA in all 61 strains of M. tuberculosis complex and 47 strains of nontuberculous mycobacteria except Mycobacterium gordonae (19). This operon is apparently absent in the syntenic region of the M. gordonae genome (Fig. 2D and E). Furthermore, the identity of the M. gordonae homolog (gene ID: 2930227449) closest to M. smegmatis Cfa was only 30%, implying that it is unlikely to be the ortholog. These observations together suggest the potential role of Cfa in TBSA synthesis, but its enzyme activity has not been demonstrated, and its *in vivo* role has not been genetically tested in mycobacteria. Further, any physiological function of Cfa in mycobacterial cells remains unknown.

### Cfa is a SAM-dependent methyltransferase.

**FIG 3:**
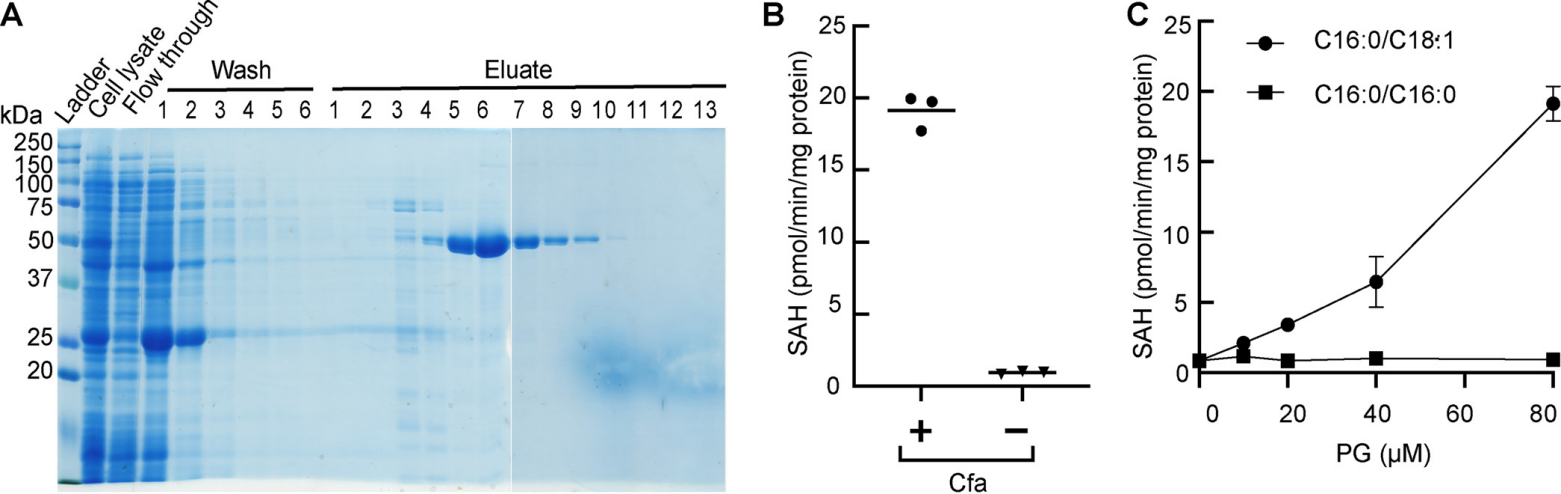
SAM-dependent enzyme activity of Cfa. (A) Purification of Cfa-6xHis by affinity column chromatography. Crude cell lysate, flowthrough, washes, and eluates were analyzed with SDS-PAGE and stained with Coomassie blue. Cfa-6xHis (expected molecular weight, 52.18 kDa) was prepared from eluate fractions 5 through 9. (B) Cfa-6xHis-dependent production of SAH in the presence of 80 μM PG C_16:0_/C_18:1_. (C) Kinetics of Cfa reaction. The rate of the SAH product formation increased with increasing concentrations of PG C_16:0_/C_18:1_ substrate. PG C_16:0_/C_16:0_ was ineffective as a substrate. All enzymatic assays were performed in triplicate.

We first tested the suggested SAM-dependent methyltransferase activity of Cfa. We cloned *cfa* into an expression vector, produced with a polyhistidine tag (Cfa-6×His) in E. coli, and purified the protein to near homogeneity by affinity chromatography (Fig. 3A). We combined eluates 5 to 9 and used the combined eluates as a source of purified enzyme. To test the enzyme activity, we incubated the enzyme with SAM and phosphatidylglycerol (PG) carrying palmitic acid (C_16:0_) at *sn*-1 and oleic acid (C_18:1_) at *sn*-2 as acyl moieties (designated PG C_16:0_/C_18:1_). The SAM-dependent enzyme activity was measured by the production of *S*-adenosyl-l-homocysteine (SAH) using a commercially available methyltransferase assay system. As shown in Fig. 3B, we detected a robust production of SAH from SAM only when the purified enzyme was added to the reaction mixture containing SAM and PG C_16:0_/C_18:1_. To test the substrate specificity, we next compared PG C_16:0_/C_18:1_ with PG C_16:0_/C_16:0_, which does not carry an oleic acid moiety. While the enzyme activity increased with increasing concentrations of PG C_16:0_/C_18:1_, PG C_16:0_/C_16:0_ did not serve as a substrate at all concentrations tested (Fig. 3C). Taken together, our data support the previous prediction that Cfa is a SAM-dependent methyltransferase that utilizes a lipid containing an oleic acid moiety as a substrate.

### Cfa detectably alters PIMs.

**FIG 4:**
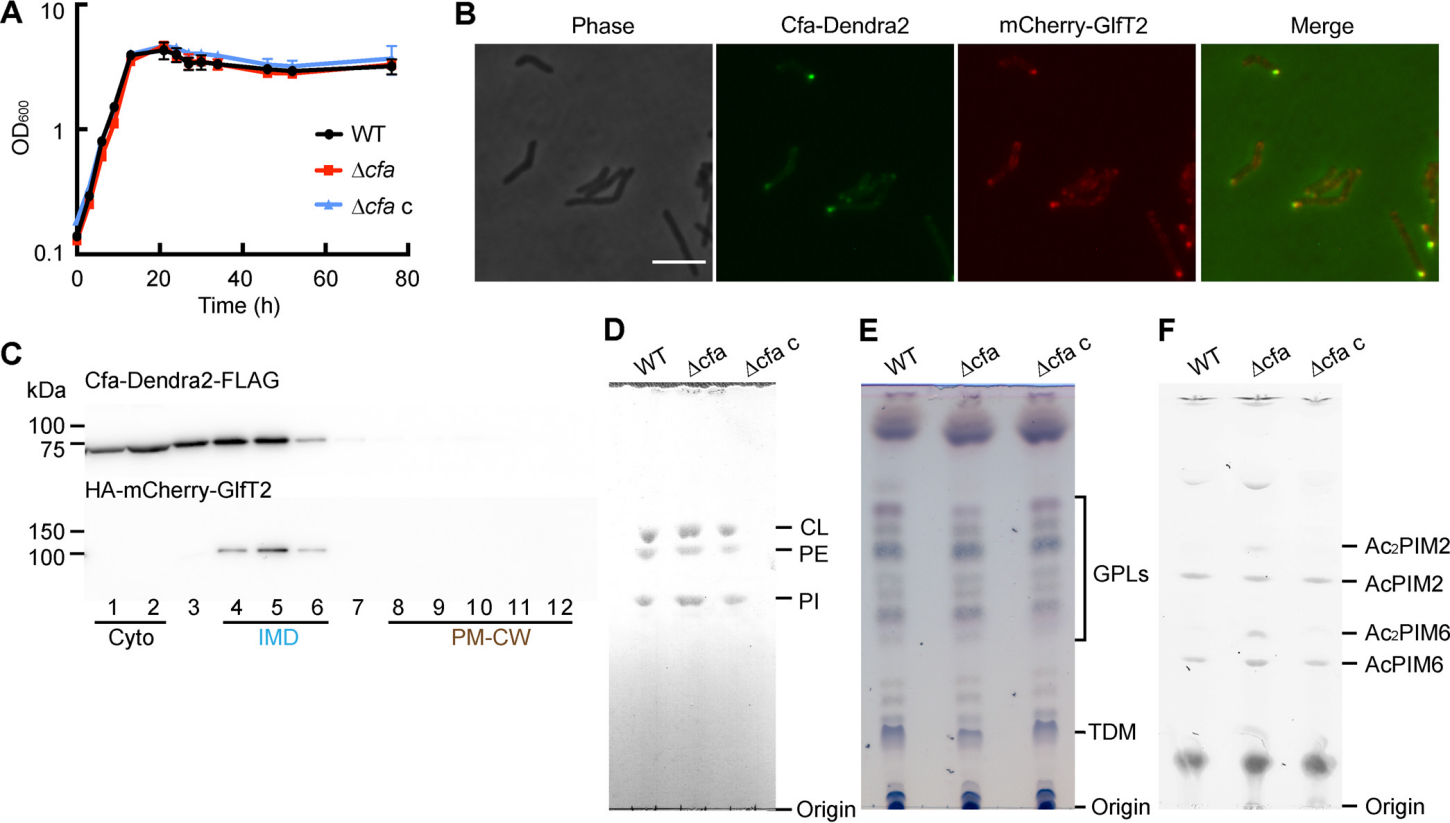
Phenotype of Δ*cfa* and subcellular localization of Cfa. (A) Growth curve for each strain grown at 37°C in Middlebrook 7H9 medium, measured by OD_600_. WT, wild type. (B) Fluorescence microscopy of the IMD marker strain, expressing Cfa-Dendra2-FLAG from the *attB* site of mycobacteriophage L5 and the IMD marker mCherry-GlfT2 from the endogenous locus. Scale bar = 5 μm. (C) Density gradient fractionation of a cell lysate of the IMD marker strain producing Cfa-Dendra2-FLAG. Cyto, cytoplasm. Anti-hemagglutinin (HA) antibody was used to detect mCherry-GlfT2, which has an N-terminal HA epitope tag (8). (D) HPTLC analysis of phospholipids visualized by molybdenum blue staining. CL, cardiolipin; PE, phosphatidylethanolamine; PI, phosphatidylinositol. (E) HPTLC analysis of glycopeptidolipids (GPLs) and trehalose dimycolate (TDM) visualized by orcinol staining. (F) HPTLC analysis of PIMs visualized by orcinol staining. HPTLC bands were assigned based on our previously published analyses (9, 56, 65, 66).

To determine the *in vivo* function of *cfa*, we next obtained a Δ*cfa* mutant from the Mycobacterial Systems Resource and complemented it with an expression vector for Cfa-Dendra2-FLAG (Δ*cfa* L5::*cfa-dendra2-flag*, or Δ*cfa* c for short). When grown in a standard rich medium (Middlebrook 7H9) without dibucaine stress, the mutant did not show any significant growth defects (Fig. 4A). Cfa was previously identified as an IMD-associated protein by high-throughput proteomic and fluorescence microscopy analyses (8, 20). Indeed, under fluorescence microscopy, Cfa-Dendra2-FLAG was specifically enriched in the subpolar region with sidewall patches (Fig. 4B), suggesting the IMD association of the protein. In our previous study, native Cfa protein was lost from the IMD proteome when the IMD membrane vesicles were purified by immunoprecipitation (8), potentially implying weak association with the IMD. Consistent with this observation, Cfa-Dendra2-FLAG was identified in both the IMD and cytosolic fractions by density gradient fractionation analysis (Fig. 4C).

Next, we extracted lipids and analyzed them with high-performance thin-layer chromatography (HPTLC). Major membrane phospholipids (Fig. 4D), including PI, PE, and cardiolipin (CL), as well as glycopeptidolipids, and trehalose dimycolate (Fig. 4E), were equivalent in density after *cfa* knockout. Interestingly, tetraacylated species of PIMs, Ac_2_PIM2 and Ac_2_PIM6, increased after *cfa* knockout (Fig. 4F). This outcome of altered PIM pools after gene deletion matches the separately observed response of PIMs to membrane fluidizers (6, 14). Combined, these results, as well as the emergence of *cfa* from a membrane fluidizer-based screen (see Fig. 2A), provided a hint for a candidate function of Δ*cfa* in control of membrane fluidization in some way that involves membrane lipids.

### Global lipidomics of *cfa* mutants.

**FIG 5:**
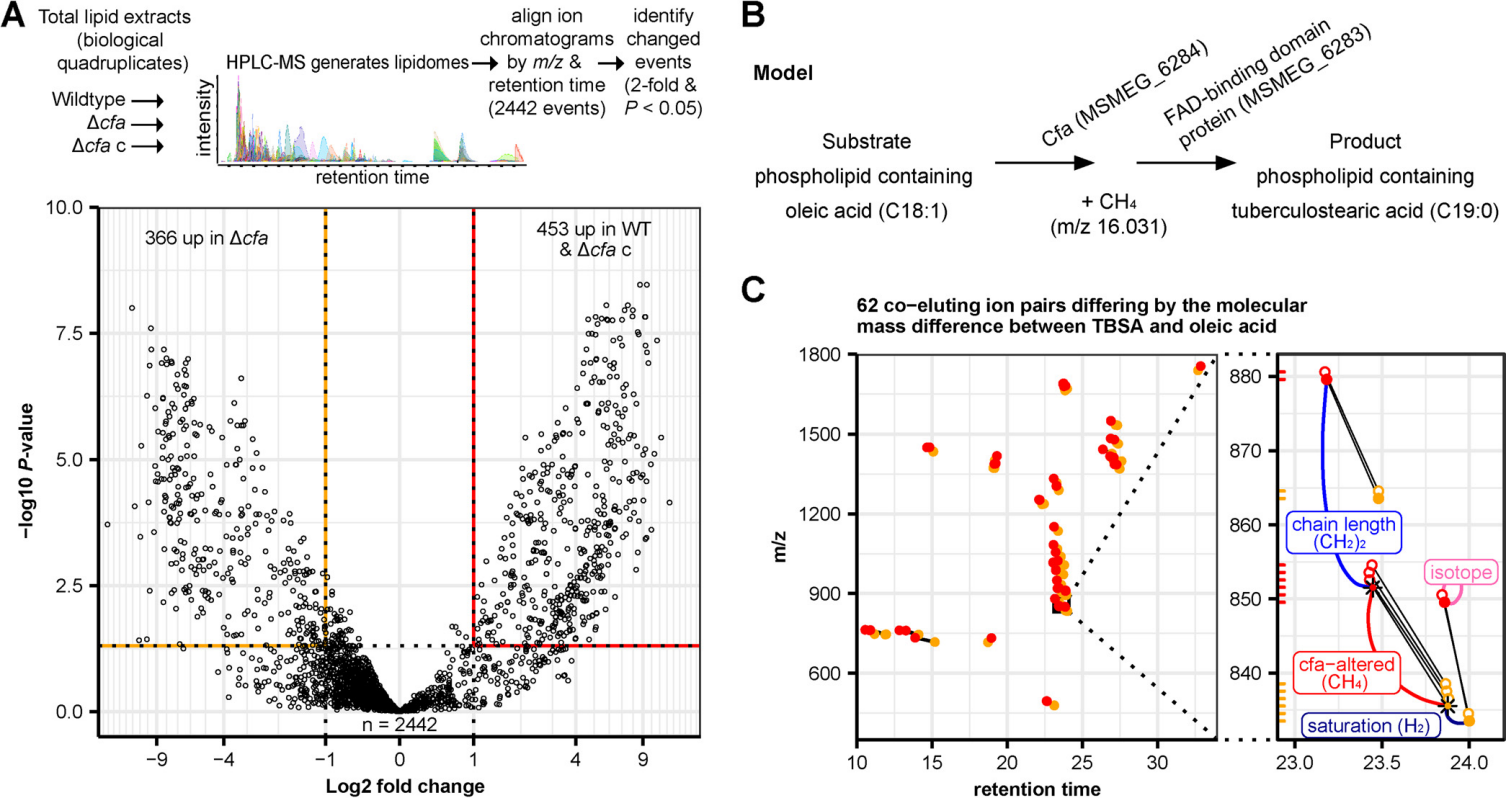
Comparative lipidomes from wild-type M. smegmatis, Δ*cfa*, and its genetic complement, indicating the absence of TBSA in Δ*cfa*. (A) Schematic of a lipidomic comparison and volcano plot of lipid ions differentially accumulating in Δ*cfa* versus the wild type and Δ*cfa* c detected by negative-mode HPLC-MS. Open circles indicate individual ions. (B) Model for Cfa enzymatic function consistent with the observed mass differences. (C) Mass versus retention time for 62 nearly co-eluting lipid pairs identified based on a CH_4_ mass change. Red circles indicate lipids enriched in the wild type and Δ*cfa* c, while orange circles indicate lipids enriched in Δ*cfa*. Lipid pairs differing by a CH_4_ mass change and <1.5-min retention time are connected by line segments. Inset, expansion of the plot area indicated by the black rectangle in the main plot showing mass values matching an [M-H]^–^ of C_19:0_, C_16:0_ PI (observed *m/z* 851.5653; red closed-over asterisk with red label), and its cognate C_18:1_, C_16:0_ PI pair (observed *m/z* 835.5333; orange closed-over asterisk). Pairs of a chain length variant, an unsaturation variant, and isotopes (open) are labeled. Mass intervals are also shown by a *y* axis marginal rug plot.

TBSA is a major fatty acid among M. smegmatis phospholipids (21, 22); in particular, PIMs exclusively carry TBSA at the *sn*-1 position of glycerol (23). If Cfa is involved in TBSA synthesis, the lack of *cfa* will result in changes in fatty acid composition, which may not be detected by low-resolution HPTLC analysis of bulk lipids. We therefore conducted an unbiased high-performance liquid chromatography mass spectrometry (HPLC-MS)-based analysis of total lipid extracts from the wild type, Δ*cfa*, and Δ*cfa* c in biological quadruplicate. This lipidomics platform broadly measures named phospholipids, neutral lipids, and many hundreds of unnamed lipids that can be tracked across genetically altered samples based on their accurate mass retention time values (Fig. 5A). Among 2,442 identified ion chromatograms, 366 ions were significantly (corrected *P* < 0.05; fold change, >2) enriched in Δ*cfa*, while 453 were enriched in wild type and Δ*cfa* c. Thus, in contrast to the narrow spectrum of PIM changes seen with HPTLC, the more sensitive MS-based lipidomics approach demonstrated a broader scope of lipidic change. These many lipid changes represented a potential explanation for the observed effects of *cfa* on recovery from membrane disorder, as well as an opportunity to discover *cfa* as a putative lipid-modifying gene.

### Comparative lipidomics query for loss of TBSA.

To identify the lipids for which *cfa* is essential, we sought targeted analysis of lipids downregulated by *cfa* deletion. First, we formulated a lipidomic query based on the arithmetic difference between the mass of oleic acid (*m/z* 282.256) and TBSA (*m/z* 298.287), which are the proposed precursors and products, respectively, of Cfa. If Cfa is essential for TBSA (C_19:0_) biosynthesis, substitution with oleic acid (C_18:1_) would reduce the overall mass of any TBSA-containing lipid by CH_4_ (*m/z* 16.031) (Fig. 5B), and the two lipids would nearly coelute. Indeed, 62 lipid molecular species pairs met these criteria in the comparison of Δ*cfa* versus the wild type and Δ*cfa* c (Fig. 5C), ranging from *m/z* 450 to 1,800 and in a retention time range of 10 to 33 min. This retention time range matches the general retention time range seen for phospholipids (24). Overall, this pattern suggested broad substitution of C_18:1_ for C_19:0_ in many types of polar lipids after deletion of *cfa*.

### Identification of *cfa*-dependent lipids.

**FIG 6:**
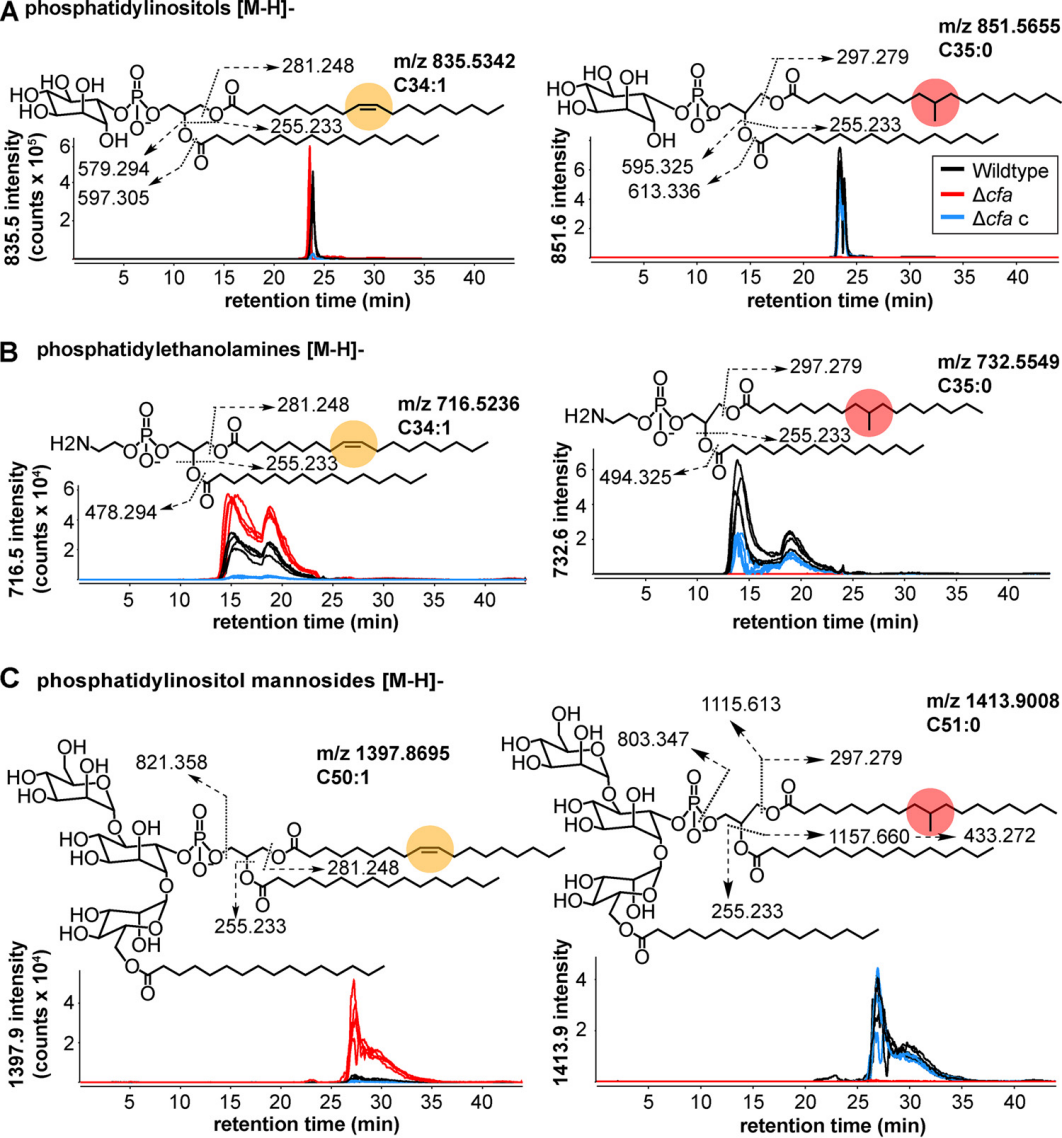
Targeted analysis of lipids regulated by *cfa*. (A) Ion chromatograms of PI containing C_18:1_, C_16:0_ (left) or C_19:0_, C_16:0_ (right) showing opposite effects of *cfa* deletion on the proposed precursor and product of Cfa. CID-MS interpreted collisional diagrams in each panel show the fragments observed by CID-MS within 10 ppm of the expected exact mass and are diagnostic for identification. Positions of fatty acid attachment, unsaturation (orange circle) and methylation (red circle) are inferred from the literature. (B) Ion chromatograms of PE. (C) Ion chromatograms of AcPIM2.

Next, we sought to identify key *cfa*-dependent lipids from these ion pairs. The M. smegmatis lipidome is not yet annotated, but we could use mass-based annotations for lipids shared with the M. tuberculosis lipidome (24, 25) and confirm chemical structures with collision-induced dissociation mass spectrometry (CID-MS). Given the large number of lipids affected by *cfa* deletion, we simplified the analysis by taking advantage of the phenomenon whereby structurally similar lipids cluster into groups with similar mass and retention time (Fig. 5B and C). Then, we implemented a strategy to chemically solve one compound in each cluster based on embedded mass values and then deduce the structures corresponding to all ions in one group. For example, one member of a *cfa*-dependent ion pair matched the expected mass of PI (*m/z* 851.566) and comprised TBSA and palmitic acid (PI C_19:0_/C_16:0_). This lipid clustered together with a PI compound matching the mass of a chain length variant (PI C_19:0_/C_18:0_), a saturation variant (PI C_19:0_/C_16:1_), and 6 isotopes of these molecules (Fig. 5C, inset). We ruled in the PI structure with CID-MS that detected glyceryl-inositol-phosphate and the defining fatty acyl fragments (Fig. 6A). The strong signal of PI C_19:0_/C_16:0_ in ion chromatograms (~600,000 counts) was consistent with the known high abundance of this PI species compared with other PI species (8, 26, 27) (Fig. 6A). Ion chromatograms also demonstrated the complete loss of PI C_19:0_/C_16:0_ in Δ*cfa* and its restoration through complementation, establishing the essential role of *cfa* in its biosynthesis. Importantly, while *cfa* was essential for the TBSA-containing form of PI, deletion increased the production of PI formed from C_18:1_ oleic acid, which is the putative precursor of TBSA (Fig. 6A). All outcomes could be explained if *cfa* encodes the essential enzyme for converting C_18:1_ oleic acid to C_19:0_ TBSA.

Extending this analysis of putative *cfa*-dependent ion pairs to other major membrane phospholipids, other *cfa*-regulated lipid pairs were identified as PE (*m/z* 716.5326) and AcPIM2 (*m/z* 1397.8695) by CID-MS (Fig. 6B and C). Similar to PI species, the *cfa* dependence of ion chromatograms of PE and AcPIM2 variants containing C_19:0_ fatty acids was complete and genetically complementable, demonstrating an essential role for *cfa*. Also, both PE and AcPIM2 showed strong increases in forms containing C_18:1_ fatty acids, which ruled out the possibility of a general block of PE and PIM biosyntheses and instead indicated the defect in the biosynthesis of TBSA-containing lipids. We note that PI C_34:1_ and PE C_34:1_ were depleted in Δ*cfa* c (Fig. 6A and B). We speculate that the complementation resulted in an overproduction of Cfa, leading to the depletion of PI and PE carrying C_18:1_. In summary, the deletion of *cfa* is associated with a selective defect in TBSA incorporation into many mycobacterial lipid families (Fig. 5 and 6), including at least three major membrane phospholipids, while leaving the larger total pools of these phospholipids containing other fatty acids intact (Fig. 4D and F).

### Effects of *cfa* deletion on mycobacterial growth and membrane integrity.

**FIG 7:**
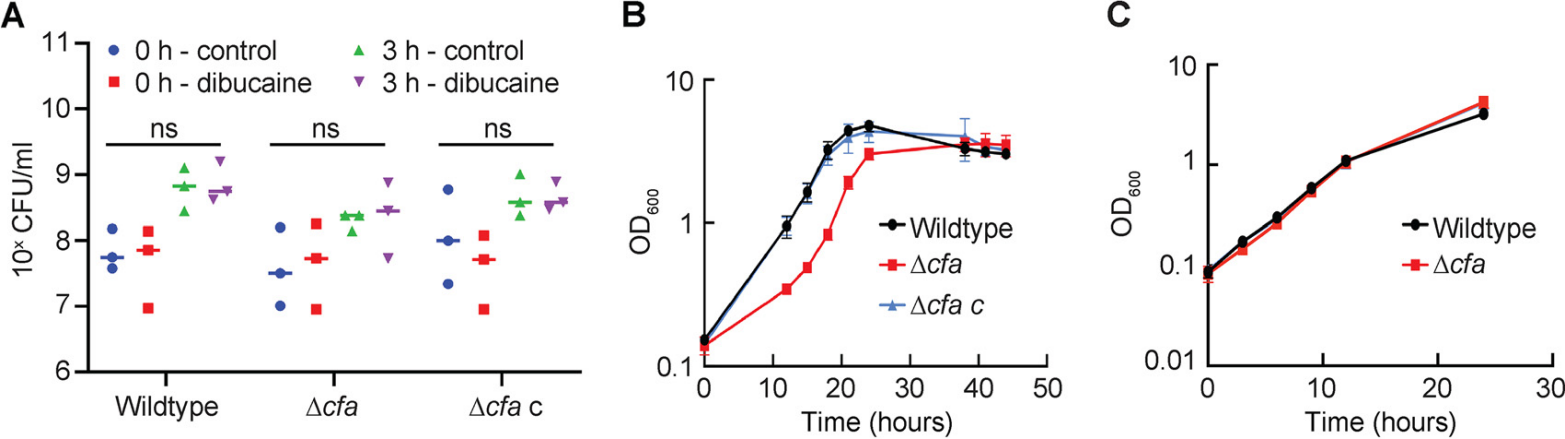
Δ*cfa* shows delayed recovery after dibucaine treatment. (A) CFU were calculated in biological triplicate to determine the survival of Δ*cfa* during dibucaine treatment. There were no statistically significant differences (indicated as ns) as determined by two-way analysis of variance (ANOVA) followed by Tukey’s multiple-comparison test. (B and C) Growth after washing out dibucaine (B) or benzyl alcohol (C) was monitored by OD_600_.

To determine the physiological function of Cfa in live cells, we next tested if the mutant is more susceptible to dibucaine treatment. We treated Δ*cfa* with 200 μg/mL dibucaine for 3 h and examined the viability of the mutant. Δ*cfa* did not show any increased sensitivity to dibucaine, and CFU did not decline from pre- to post-treatment (Fig. 7A). We next examined the recovery after dibucaine treatment in a pulse-chase experiment and found that the growth rate of Δ*cfa* was slower than that of the wild type initially, but the mutant recovered during culture in dibucaine-free media after 15 to 18 h (Fig. 7B). Thus, Δ*cfa* is defective in recovering from, but not in surviving during, dibucaine treatment under the conditions tested. We tested another membrane fluidizer, benzyl alcohol, and found that Δ*cfa* was not defective in recovering from this membrane fluidizer (Fig. 7C), suggesting that the effect of dibucaine on the mycobacterial plasma membrane is distinct from that of benzyl alcohol.

**FIG 8:**
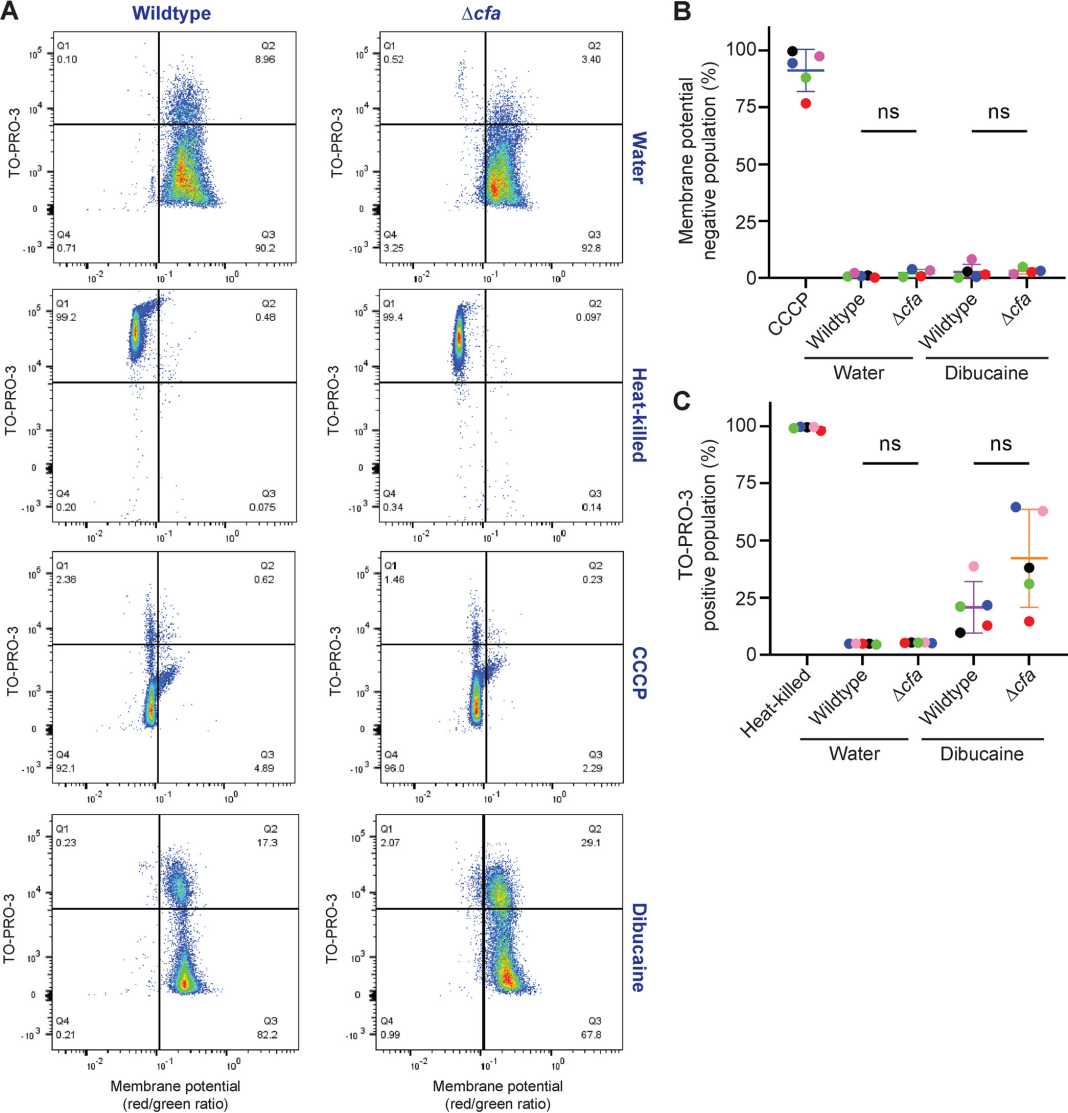
Membrane permeability barrier and proton gradient formation are largely unaffected in Δ*cfa*. (A) Wild-type or Δ*cfa* bacteria were stained with a membrane potential sensor [DiOC_2_(3)] and a membrane permeability sensor (TO-PRO-3) with or without dibucaine. Membrane potential was assessed by the fluorescence ratio (red/green) of DiOC_2_(3). Carbonyl cyanide *m*-chlorophenyl hydrazone (CCCP) was used to disrupt the membrane potential. Heat (65°C, 1 h) was used to permeabilize the membrane. The data set is a representative result from 5 experiments. (B and C) A population of wild-type or Δ*cfa* cells that were negative for membrane potential [measured by the fluorescence ratio (red/green) of DiOC_2_(3)] (B) or positive for membrane permeability (measured by TO-PRO-3 stains) (C) was plotted. Each color shows a set of data from a single replicate. Statistical significances were determined by the Kruskal-Wallis test, followed by Dunn’s multiple comparison test. ns, not statistically significant.

Depletion of TBSA and accumulation of *cis*-monounsaturated fatty acids in membrane phospholipids predict increased membrane fluidity, which could explain the slower recovery from membrane-fluidizing stress. At the extreme, excess membrane fluidity can compromise the permeability barrier such that compounds can orthogonally transit through the plasma membrane (28, 29). To test if the plasma membrane of Δ*cfa* is more permeable, we used the membrane-impermeable DNA staining dye TO-PRO-3 and the membrane potential probe 3,3′-diethyloxacarbocyanine iodide [DiOC_2_(3)]. As a positive control, heat-killed cells became TO-PRO-3 positive and lost membrane potential (Fig. 8A). In contrast, cells lost membrane potential upon treatment with carbonyl cyanide *m*-chlorophenyl hydrazone (CCCP), a membrane potential uncoupler, while they remained TO-PRO-3 negative (Fig. 8A). We compared the wild type and Δ*cfa* with or without dibucaine treatment in five experiments, which reproducibly found that both the wild type and Δ*cfa* maintained the membrane potential even after dibucaine treatment (Fig. 8A and B). While subpopulations (10 to 60%) of both wild-type and mutant cells became TO-PRO-3-positive after dibucaine treatment (Fig. 8A and C), there was no statistically significant difference between the strains (Fig. 8C). With these data taken together, Δ*cfa* is defective in recovering growth after membrane-fluidizing treatments, but plasma membrane integrity is not substantially compromised to allow orthogonal transit of measured molecules.

### Membrane domain partitioning is altered by *cfa* deletion.

**FIG 9:**
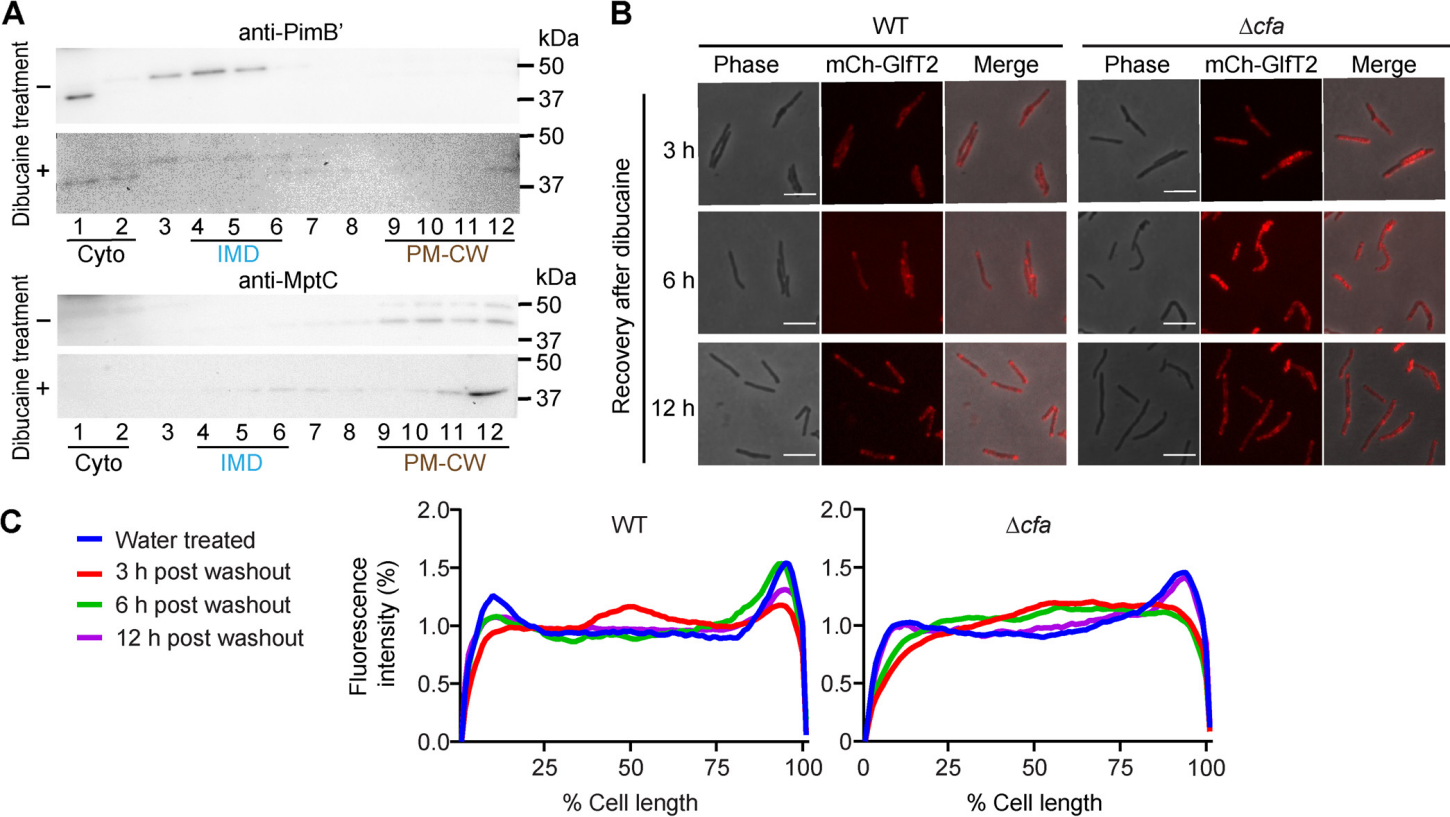
Δ*cfa* is defective in restoring subpolar membrane partitioning. (A) Western blotting of density gradient fractions of Δ*cfa* cell lysates. Before or after 3-h dibucaine treatment, log-phase cells were lysed and fractionated by sucrose density gradient sedimentation. PimB′, an IMD marker; MptC, a PM-CW marker. (B) Recovery of the subpolar localization of the IMD marker mCherry-GlfT2 (mCh-GlfT2) expressed from the endogenous locus in wild-type (WT) or Δ*cfa* cells. Cells were treated with 200 μg/mL dibucaine for 3 h, washed, and recovered for up to 12 h. Images were taken at 3, 6, and 12 h. Scale bar = 5 μm. (C) Profiles of relative fluorescence intensities from images taken as in panel B. Note that the wild-type cells are in a late log phase by 12 h post-dibucaine washout: the reduced level of subpolar enrichment at 12 h is likely due to the shift in growth phase, as reported before (13). Wild type: *n* = 46 (water), 93 (3 h), 54 (6 h), or 92 (12 h). Δ*cfa*: *n* = 75 (water), 205 (3 h), 138 (6 h), or 77 (12 h).

Our results so far showed that, in the absence of dibucaine challenge, Δ*cfa* is not overtly defective in growth or its plasma membrane integrity. Since Δ*cfa* was initially identified as a mutant sensitive to dibucaine, which alters membrane compartmentalization, we wondered if Δ*cfa* is defective in recovering from dibucaine-induced disruption of membrane partitioning. Biochemically, both the IMD and PM-CW were purified from Δ*cfa* under normal growth conditions (Fig. 9A). However, after 3 h of dibucaine treatment, the IMD marker PimB′ became diffuse and less enriched in the IMD fractions (Fig. 9A) like the wild type (see Fig. 1D). We also observed more diffuse distribution of the PM-CW protein MptC spanning to the lighter density region (Fig. 9A), compared with the wild type (see Fig. 1D), suggesting more severe impact of dibucaine on membrane partitioning in Δ*cfa*. We then examined by microscopy the recovery of the subpolar IMD enrichment over time after dibucaine treatment. In wild-type cells, subpolar enrichment of the IMD marker mCherry-GlfT2 was partially restored after a 3-h recovery and fully restored after 6 h (Fig. 9B and C). In contrast, for Δ*cfa*, the subpolar enrichment of mCherry-GlfT2 required 12 h for full recovery (Fig. 9B and C). Thus, Δ*cfa* is delayed in recreating subpolar membrane partitioning after dibucaine treatment.

## DISCUSSION

Tuberculostearic acid (TBSA) has been studied for decades as an abundant Mycobacterium-characteristic lipid. TBSA is produced by most mycobacterial species, where it amounts to 10 to 20% of fatty acids (30, 31). Because TBSA is not produced by humans, it has been proposed as a diagnostic test for tuberculosis (32–38). The structure of TBSA was determined in 1934 (39), and it was first synthesized in 1947 (40). TBSA biosynthesis from oleic acid was proposed in 1962 (41), which frames the interpretations of the cellular lipidomics performed here.

There are seven and nine paralogs of *cfa* in M. smegmatis and M. tuberculosis, respectively, and at least two other genes have also been proposed to mediate the biosynthesis of TBSA in mycobacteria (42, 43). Our study shows that *cfa* (*MSMEG_6284* in M. smegmatis), rather than the other two previously proposed genes, encodes the SAM-dependent methyltransferase needed for the biosynthesis of many TBSA-containing lipids, including three major families of membrane phospholipids: PI, PE, and PIMs (23, 32, 44). The defects of Δ*cfa* that are restored with *cfa* complementation unequivocally demonstrate the necessary and sufficient role of Cfa in TBSA synthesis. Given the prior work by Machida et al. (18), we speculate that the FAD-binding domain protein is the oxidoreductase, which mediates the second step of the reaction, where *cis*-9,10-methylene octadecanoic acid produced by Cfa is reduced to TBSA.

Subcellular location of Cfa remains an open question. Prior studies suggested that Cfa is an IMD-associated protein (8, 20), and our fluorescence microscopy analysis supported this conclusion. However, immunoblot analysis of sucrose density gradient fractions suggested that Cfa may not be strictly IMD-associated. We have a few examples of such proteins (e.g., MSMEG_0949 and MSMEG_2329), which were identified as IMD-associated proteins based on fluorescence microscopy and proteomics and were cytoplasmic based on density gradient fractionation (45). Since Cfa binds both SAM, a soluble substrate, and oleic acid, a lipidic substrate, there may be physiological reasons why Cfa is found in both cytoplasm and the IMD. However, we acknowledge that the localization of Cfa reported in this study may be affected by the attachment of Dendra2, a large fluorescent protein. Additionally, whereas we successfully used PG C_16:0_/C_18:1_ as a Cfa substrate in an enzymatic assay, we do not know if the *in vivo* substrates of Cfa are phospholipids such as PG or biosynthetic intermediates such as oleoyl-CoA, which may be available in the cytoplasm bound by an acyl-CoA binding protein. Additional studies are needed to determine if and how Cfa associates with the plasma membrane, how it catalyzes the first step of TBSA biosynthesis, how the membrane association contributes to its catalytic activity, and how synthesized TBSA distributes to both the IMD and PM-CW.

Through comparative lipidomics, unbiased examination of all ionizable lipids altered through gene deletion in live mycobacterial cells is now possible (46). Using this platform, we revealed a dominant pattern of CH_4_ loss among polar lipids in Δ*cfa*. Mechanistically, we strongly favor the interpretation that *cfa* deletion causes loss of these lipids through ablation of the shared TBSA pool, rather than some adjunct effect of Cfa on overall PI, PE, and PIM synthesis. Lipidomics data demonstrated both the ablation of TBSA-containing phospholipids and increased C_18:1_-containing PE, PI, and AcPIM2. The opposite effects of *cfa* deletion based on the fatty acid present ruled out a general block in synthesis of these phospholipid products, and it strongly supports that *cfa* deletion ablated the downstream product (TBSA) and increased the immediate upstream product (oleic acid). Also, the opposite regulation of distinct PI and PE acyl forms by *cfa* can resolve the otherwise apparent contradiction between preserved total PI and PE pools observed in HPTLC and the complete loss of TBSA-containing PI and PE in MS-based lipidomics.

In Mycobacterium phlei, the fatty acid composition shifts from TBSA to *cis*-unsaturated fatty acids such as oleic and linoleic acids when grown at a lower temperature (31). This observation implies that tilting the balance to *cis*-unsaturated fatty acid from TBSA helps the cells to maintain membrane fluidity under a colder growth temperature, where the membrane becomes more ordered. Therefore, TBSA may be involved in homeoviscous adaptation, a stress response mechanism (47, 48), in which TBSA makes the plasma membrane more rigid than *cis*-unsaturated fatty acids. Our data are consistent with the importance of TBSA in homeoviscous adaptation as the lack of TBSA and the accumulation of oleic acids in Δ*cfa* would make the mutant’s plasma membrane more disordered, explaining the reason why Δ*cfa* is more vulnerable to dibucaine, a membrane-fluidizing molecule. We propose that TBSA biosynthesis is important for creating a lipid environment resilient to external threats that increase the fluidity of the mycobacterial plasma membrane.

Branched-chain fatty acids are important for membrane homeostasis, and their depletion makes bacterial cells defective in membrane fission during L-form proliferation and fluidity maintenance (49–51). While these studies used iso and anteiso fatty acids in other bacteria, we propose that TBSA in mycobacteria could similarly support membrane integrity. However, our data indicate that the defect in synthesizing TBSA correlates with the slower kinetics of membrane domain partitioning and not with other functions. First, Δ*cfa* grows like the wild type, indicating that there are no gross impacts on growth under laboratory culture conditions. Second, we used the membrane potential probe DiOC_2_(3) and demonstrated that Δ*cfa* can create a proton gradient across the plasma membrane for oxidative phosphorylation. Third, we tested the membrane permeability of Δ*cfa* using the fluorescent DNA-binding dye TO-PRO-3 and did not observe any significant defects relative to the wild type with or without dibucaine challenge. These observations suggested that the lack of Cfa and the resultant changes in the ratio of oleic acid to TBSA do not have significant effects on overall membrane integrity. In contrast, we found that there was a delay in subpolar IMD enrichment in Δ*cfa* during recovery from dibucaine treatment. This delay coincided with delayed recovery of growth after dibucaine treatment. Benzyl alcohol has a similar disruptive effect on membrane partitioning (15), and yet the recovery of growth of Δ*cfa* after the treatment with this membrane fluidizer was no different from that of the wild type. Dibucaine has been suggested to insert into the ordered membrane domain preferentially, while benzyl alcohol preferentially inserts into disordered regions (16, 52). Thus, we speculate that the molecular mechanisms of disrupting mycobacterial membrane partitioning are different between these two membrane fluidizers. Although TBSA-containing phospholipids are distributed in both the IMD and the PM-CW (8), they could have more dominant roles in maintaining the integrity of the PM-CW, which, we speculate, is more ordered than the IMD (53). Being consistent with this model, we observed changes in subcellular fractionation patterns of not only IMD but PM-CW proteins in Δ*cfa* upon dibucaine challenge. Overall, our results, combined with known roles of branched-chain fatty acids, support the critical role of TBSA in membrane partitioning. Nevertheless, we acknowledge a less likely scenario that the membrane partitioning defect of Δ*cfa* is not a direct consequence of the deficient TBSA biosynthesis. Determining the precise mechanisms of how TBSA controls membrane partitioning at the molecular level is an important direction for future research.

We recently reported inositol acylation of PIMs as a response to membrane fluidization conserved in M. tuberculosis (6). Our current study implies a potential role of TBSA-containing lipids, which are widely conserved in mycobacteria, including M. tuberculosis, in homeoviscous adaptation. One transposon mutagenesis study predicts *cfa* to be essential in M. tuberculosis (54). Furthermore, the *cfa* ortholog in Mycobacterium bovis BCG is critical for persistence during passage in bovine lymph node (55). These studies illuminate the potential importance of homeoviscous adaptation in M. tuberculosis, an obligate human pathogen that does not experience temperature fluctuations. Thus, our studies suggest the intriguing hypothesis that there are yet-to-be-defined host factors that affect the pathogen’s membrane fluidity during host-pathogen interactions.

## MATERIALS AND METHODS

### Growth and treatment with membrane fluidizers.

M. smegmatis mc^2^155 was grown at 37°C in Middlebrook 7H9 broth supplemented with 11 mM glucose, 14.5 mM NaCl, and 0.05% (vol/vol) Tween 80. Dibucaine (Sigma-Aldrich) was added to a log-phase culture at the final concentration of 200 μg/mL, and an equivalent volume of water was used as a vehicle control. After a 3-h incubation at 37°C, the culture was washed with phosphate-buffered saline (PBS) containing 0.05% Tween 80 (PBST) three times and resuspended in the same volume of Middlebrook 7H9 broth for recovery. Benzyl alcohol treatment was identical to dibucaine treatment except that the treatment was for 1 h at the final concentration of 100 mM, as described previously (14). CFU were determined by serially diluting cell culture using Middlebrook 7H9 broth and spotting 5 μL on Middlebrook 7H10 agar supplemented with 11 mM glucose and 14.5 mM NaCl. The agar plates were incubated at 37°C for 2 to 3 days before the number of microcolonies was determined.

### Transposon sequencing.

We prepared a transposon mutant library using ΦMycoMarT7 phage as before (15), grew the library of cells to a log phase, and treated the cells with dibucaine (or water as the vehicle control) for 3 h. The cells were washed to remove dibucaine and recovered in Middlebrook 7H9 broth for ~16 h. Genomic DNA was purified, sheared, and barcoded. Transposon insertion sites were then amplified by a nested PCR. To prepare the library for high-throughput DNA sequencing, we used the KAPA library preparation kit (Kapa Biosystems) and TruSeq adapters (Illumina) as before (56). The library was sequenced by 100-bp paired-end sequencing using the Illumina HiSeq 3000 platform. Identified genes were compared between water- or dibucaine-treated samples using TRANSIT as described in the literature (57, 58). Library sequencing yielded 5 million unique transposon-inserted-sequences, which covered over 35% of the possible TA sites in the genome.

### Bioinformatic analysis.

Cfa proteins from M. smegmatis mc^2^155, M. marinum, *M. chlorophenolicum*, M. abscessus, M. tuberculosis H37Rv, M. avium, and M. leprae were aligned using UniProt Align (59). For the analysis of the M. gordonae genome region, the genome region of M. gordonae strain X7091 chromosome (GenBank sequence ID: CP070973.1; region 6782643 to 6796036) and that of M. smegmatis mc^2^155 chromosome (GenBank sequence ID: CP000480.1; region 6340496 to 6353816) were aligned using NCBI Nucleotide BLAST. To identify M. gordonae Cfa protein homolog (ID:2930227449), M. smegmatis Cfa was subjected to a BLAST search (Protein BLAST) against the genome of M. gordonae strain X7091 (available from the JGI Integrated Microbial Genomes and Microbiomes portal; https://img.jgi.doe.gov/).

### Cfa purification and methyltransferase assay.

We amplified the *cfa* gene from M. smegmatis genomic DNA using a forward primer (5′-CAC CGC ATC CAT GAC CAC ATT CAA AGA ACG CGA GAC GTC CAC AGC GG-3′) and a reverse primer (5′-GGC GGA CCA CCA GGG CCG CA-3′) and cloned into pET101 TOPO vector using the Champion pET101 directional TOPO expression kit (Invitrogen). The plasmid was introduced into E. coli BL21 (Invitrogen). The transformed E. coli strain was grown at 30°C for 6 h, and the production of Cfa-6×His was induced for 6 h with 1 mM isopropyl β-d-1-thiogalactopyranoside (Fisher). Cells were washed, resuspended in a lysis buffer containing 1 mg/mL lysozyme (Fisher), 50 mM HEPES-NaOH (pH 7.4), 200 mM NaCl, 0.33 mM phenylmethanesulfonyl fluoride, 0.33 mM dithiothreitol, and lysed by sonication. The lysate was centrifuged to remove cell debris, and the supernatant was applied to 250 μL bed volume of Nickel NTA agarose resin (GoldBio) for affinity column chromatography. The crude lysate was loaded onto the column, and the flowthrough was collected. The resin was washed twice with 1 mL of wash buffer (100 mM HEPES-NaOH [pH 7.5], 500 mM NaCl, and 10 mM imidazole) supplemented with 0.05% Tween 20 and four times with 1 mL of wash buffer (without Tween 20). The bound protein was eluted twice with 200 μL of 100 mM HEPES-NaOH (pH 7.5) containing 75 mM imidazole, once with 200 μL of 100 mM HEPES-NaOH (pH 7.5) containing 125 mM imidazole, and 10 times with 200 μL of 100 mM HEPES-NaOH (pH 7.5) containing 250 mM imidazole. Eluate fractions 5 to 9 were combined, loaded onto an Amicon Ultra-4 centrifugal filter unit (EMD Millipore), and washed three times using 4 mL of 20 mM HEPES-NaOH (pH 7.5).

The MTase-Glo methyltransferase assay (Promega) was used, following the manufacturer’s guidelines, to determine the enzymatic activity of Cfa. PGs, 2-oleoyl-1-palmitoyl-*sn*-glycero-3-phospho-*rac*-(1-glycerol) (PG C_16:0_/C_18:1_) and 1,2-dipalmitoyl-*sn*-glycero-3-phosphate-*rac*-(1-glycerol) (PG C_16:0_/C_16:0_), were from Sigma-Aldrich and were dissolved in 0.1% TritonX-100 at 100 mM as stock solutions. Following the manufacturer’s guidelines, a standard curve was prepared using SAH. The Cfa enzyme (at the final concentration of 625 μg/mL) was incubated with 0 to 80 μM PG and 10 μM SAM for 30 min at 37°C. The produced SAH was quantified, following the manufacturer’s guidelines, by incubation with MTase-Glo reagent and MTase-Glo detection solution (30 min each at room temperature), and luminescence was detected using a Synergy 2 plate reader (BioTek). All assays were performed in triplicate.

### Live cell imaging.

Cells were grown to log phase (OD_600_, 0.5 to ~1.0), and 5 or 10 μL of cell culture was placed on a 1% (wt/vol in water) agar pad on a glass slide, and fluorescent protein localization was visualized using a Nikon Eclipse E600 microscope (100× objective; numerical aperture [NA], 1.30) equipped with an ORCA-ER cooled charge-coupled-device camera (Hamamatsu) and Openlab software 5.5.2 (Improvision). All fluorescence images were taken at the exposure of 4 s with a gain of 4. Fluorescence intensity profiles were quantified as before (15). Briefly, cell shape was contoured using Oufti (60), and each cell was divided into 100 sections along the long axis. Average relative fluorescence intensity was calculated using MATLAB with published scripts (61) and plotted along the normalized cell length.

### Preparation of recombinant strains.

M. smegmatis expressing both mCherry-GlfT2 and Ppm1-mNeonGreen from their endogenous loci was previously established (8). To visualize DivIVA-eGFP, we used the same M. smegmatis strain expressing DivIVA-eGFP and mCherry-GlfT2 as before (14). M. smegmatis Δ*cfa* and a vector to express Cfa-Dendra2-FLAG were obtained from MSRdb (62). To express mCherry-GlfT2 in Δ*cfa*, pMUM053 was electroporated to replace the endogenous *glfT2* gene with an engineered *mCherry-glfT2* gene as before (8).

### Density gradient fractionation, SDS-PAGE, and immunoblotting.

Subcellular fractionation was performed as before (8, 9). Briefly, cells were grown to a log phase (OD_600_, 0.5 to ~1.0), harvested, and lysed by nitrogen cavitation. The cell lysate was placed on top of a sucrose gradient (20 to 50%, wt/vol) and fractionated by sedimentation at 35,000 rpm for 6 h on an SW-40Ti rotor (Beckman-Coulter) at 4°C. SDS-PAGE and immunoblotting of gradient fractions were as previously described (8, 45). Rabbit anti-PimB′ and anti-MptC antibodies were raised previously (63), affinity-purified, and used at 1.0 and 1.1 μg/mL, respectively. Mouse anti-FLAG M2 antibody was from Sigma-Aldrich and used at 1 μg/mL. Horseradish peroxidase-conjugated donkey anti-rabbit (Cytiva) or sheep anti-mouse antibody (Sigma-Aldrich) was used at a 4,000× dilution as a secondary antibody, and the protein bands were visualized by chemiluminescence. Ppm1-mNeonGreen was visualized by in-gel fluorescence imaging. Both luminescence and fluorescence were detected using either an ImageQuant LAS 4000mini or an Amersham ImageQuant 800 system (Cytiva).

### Lipid extraction, HPTLC, and lipidomics.

In quadruplicates, each strain was harvested at a log phase (OD_600_, 0.5 to ~1.0), and the wet cell pellets were sequentially treated with 20 volumes of chloroform/methanol (2:1, vol/vol) relative to cell mass (i.e., 20 mL per g wet pellet), 10 volumes of chloroform/methanol (1:1, vol/vol), and 10 volumes of chloroform/methanol (1:2, vol/vol). A 10% volume of the combined organic phase was set aside for HPTLC analysis, and the remainder was subjected to lipidomic analysis. For HPTLC analysis, combined organic phase was dried under a stream of nitrogen gas, and the dried lipids were resuspended in 5 volumes of chloroform/methanol (1:1, vol/vol) relative to the original pellet weight (i.e., 5 mL per g wet pellet). Then, 10 μL of lipid extracts was spotted onto an HPTLC silica gel 60 sheet (Merck) and chromatographed using chloroform/methanol/13 M ammonia/1 M ammonium acetate/water (180:140:9:9:23, vol/vol/vol/vol/vol) as a mobile phase. PIMs were visualized by spraying the HPTLC plate with an orcinol spray reagent (16 mM orcinol, 2 M H_2_SO_4_, and 14 M ethanol in water) and baking at 120°C. Phospholipids were visualized using a molybdenum blue spray reagent (Sigma-Aldrich). For the purification of glycopeptidolipids (GPLs) and trehalose dimycolate (TDM), dried lipids were further purified by chloroform/methanol/water (8:4:3, vol/vol/vol) phase partitioning. The organic phase containing GPLs and TDM was dried under a nitrogen gas stream, and the dried lipids were resuspended in chloroform/methanol/water (9:1:0.1, vol/vol/vol). Lipids were separated on an HPTLC plate using a mobile phase containing chloroform/methanol/water (9:1:0.1, vol/vol/vol). GPLs were visualized using an orcinol spray reagent. Lipidomic analysis and CID-MS were performed as previously described (8) using an Agilent 1260 Infinity LC system and 6546 quadrupole time of flight (QTOF) mass spectrometer. Dried lipids were dissolved in hexane/isopropanol (70:30, vol/vol) at 1 mg/mL by dry weight and were separated using a normal-phase Inerstil Diol column (GL Sciences, Tokyo, Japan) with 0.1% formic acid and 0.05% aqueous ammonia added to all solvents. HPLC/MS data were analyzed using MassHunter (Agilent) and R for statistical analysis and data visualizations. Raw data and R code are available upon request.

### Flow cytometry.

A 2-mL aliquot of log-phase cells was treated with 200 μg/mL dibucaine for 3 h at 37°C with shaking. Cells were then treated with 100 nM TO-PRO-3 (Thermo Fisher Scientific) and 30 μM DiOC_2_(3) (TCI Chemicals). After a 15-min incubation, cells were centrifuged at 2,000 × *g* for 5 min, and the pellet was resuspended in 2% formaldehyde in PBS for fixation. Fixed cells were washed again and resuspended in PBS for flow cytometry analyses using a three-laser (405 nm, 488 nm, and 640 nm) Dual LSRFortessa instrument (BD Biosciences). As in a previous report (64), DiOC_2_(3) was excited at 488 nm with red emission detected through one filter set (a long-pass [LP] filter [600 nm] and a band-pass [BP] filter [610 nm/20 nm]), and green emission was detected through another filter set (LP filter, 505 nm; BP filter, 525 nm/50 nm). TO-PRO-3 was excited at 640 nm and detected through a BP filter (670 nm/30 nm). As a positive control for membrane permeability (TO-PRO-3 labeling), cells were heat-killed for 1 h at 65°C. As a positive control for the disruption of membrane potential [DiOC_2_(3) labeling], cells were incubated with 25 μM carbonyl cyanide *m*-chlorophenyl hydrazone (CCCP, Sigma-Aldrich) for 1 h. Data were analyzed using FlowJo 10.0 (FlowJo LLC, BD). The red/green ratio of DiOC_2_(3) was determined using the derived parameter function comparing the DiOC_2_(3) red and green median fluorescence parameters.
